# Mediating effects of sleep on mental health in older adolescents: Findings from the Burn 2 Learn randomized controlled trial

**DOI:** 10.1111/sms.14463

**Published:** 2023-08-09

**Authors:** Angus A. Leahy, Thierno M. O. Diallo, Narelle Eather, Mitch J. Duncan, Jordan J. Smith, Philip J. Morgan, David R. Lubans

**Affiliations:** ^1^ Centre for Active Living and Learning, College of Human and Social Futures University of Newcastle Callaghan New South Wales Australia; ^2^ Hunter Medical Research Institute New Lambton Heights New South Wales Australia; ^3^ School of Social Sciences Western Sydney University Penrith New South Wales Australia; ^4^ College of Health, Medicine and Wellbeing University of Newcastle Callaghan New South Wales Australia; ^5^ Faculty of Sport and Health Sciences University of Jyväskylä Jyväskylä Finland

**Keywords:** adolescent, mediation, mental health, physical activity, sleep

## Abstract

**Purpose:**

Our study explored the mediating effect of sleep‐related variables on older adolescents' mental health in the context of a school‐based physical activity intervention.

**Methods:**

We evaluated the Burn 2 Learn (B2L) intervention using a cluster randomized controlled trial, which included two cohorts. Participants for this sub‐study were from the second cohort, which included 292 older adolescents (16.0 ± 0.5 years) from 10 secondary schools in New South Wales, Australia. Teachers at intervention schools delivered two high‐intensity activity breaks (approximately 10 mins) per week to students during academic lessons. Participants completed measures of mental health (i.e., perceived stress and internalizing problems) and hypothesized mediators (i.e., sleep duration, sleep latency, awakenings, and daytime sleepiness) at baseline (February–April 2019) and post‐intervention (August–September 2019). Single mediation analyses were conducted to explore the potential mediating effects of sleep variables on mental health outcomes using a product‐of‐coefficient test.

**Results:**

We observed a small statistically significant effect for perceived stress (*β* = −0.11, SE = 0.034, *p* = 0.002), but not for internalizing problems (*β* = 0.02, SE = 0.051, *p* = 0.760). There were no significant intervention effects for sleep‐related variables. Several sleep‐related variables were associated with mental health outcomes but no mediated effects were found.

**Conclusion:**

The B2L intervention had a small beneficial effect on perceived stress, however our mediation analyses suggest this was not explained by changes in sleep‐related variables. Markers of sleep were associated with mental health constructs, highlighting the importance of sleep for good psychological health. However, in the context of a physical activity intervention, effects on mental health may be driven by other behavioral, neurobiological, or psychosocial mechanisms.

## 1 INTRODUCTION

Adolescence is a period of rapid growth, and generally considered to be a healthy period in a person's life, due to low levels of morbidity and mortality. However, adolescence is also the peak period of onset for mental disorders.^1^ Global secular trends suggest levels of stress, anxiety, and depression among adolescents have increased in recent decades.^2^ A potential cause for increased mental health issues may be due to pressures associated with academic performance (i.e., increased workload, university entrance requirements, and pressure to perform from parents and teachers). Indeed, a recent systematic review highlighted that approximately one in six students in their final 2 years of secondary schooling experience excessive distress during this critical time period.^3^ This is worrying, considering academic‐related stress can have a significant impact on adolescents' current and future health, and may undermine academic performance during a consequential period of schooling.^4^

To attain good health, the World Health Organization recommends adolescents accumulate an average of 60 min per day of moderate‐to‐vigorous physical activity (MVPA). It is also recommended that young people engage in vigorous‐intensity and muscle‐ and bone‐strengthening activity on at least 3 days per week.^5^ Globally, more than 80% of adolescents do not meet these physical activity recommendations, with the lowest levels of activity typically observed among older adolescents (i.e., 15–19 years).^5^ Schools are universally considered to be ideal settings to help promote physical activity, however, physical activity research involving older adolescents is scare.^6^ Academic‐related stress may be attenuated through increased physical activity, however there is limited opportunities during the school day for older adolescents to be active, as time is often redirected towards other academic subjects.^7^ The final years of schooling are a crucial period for shaping young people's health behaviors as older adolescents begin developing a sense of identity and independence as they transition into adulthood. Further, many adolescents are legally required to attend school, and this period of time presents the final opportunity to deliver interventions to adolescents in a structured setting. Once adolescents leave school, it is much more challenging to deliver interventions in a systematic way.

Engaging in regular aerobic MVPA is also a primary means of developing cardiorespiratory fitness (CRF), which is an important marker of current and future health.^8^ In addition to the well‐established physical health benefits,^9^ participation in physical activity of sufficient intensity and volume to develop CRF has beneficial effects for adolescents' mental health.^10^ For example, cross‐sectional evidence has demonstrated an association between CRF and mental health (i.e., improved well‐being and reduced ill‐being) in older adolescents.^11^ While there is an abundance of evidence demonstrating that physical activity is effective in promoting mental health,^10^ there is a need to understand for whom, and under what conditions, physical activity provides such improvements. Understanding the mechanisms of physical activity on mental health effects in the context of the school setting, can help optimize the impact of future interventions.

A conceptual framework proposed by Lubans and colleagues^12^ suggests there are three broad categories of mechanisms thought to underpin the effects of physical activity on mental health outcomes in youth. First, physical activity may improve mental health via its effects on neurobiological mechanisms, such as changes in brain structure and function (e.g., neurogenesis). Second, physical activity may influence mental health via a range of psychosocial factors. For example, the development of positive physical self‐perceptions and satisfying basic psychological needs for relatedness, competence, and autonomy.^12^ Finally, the behavioral mechanism hypothesis proposes that improvements in mental health may result from changes in other behaviors, such as self‐regulatory behaviors and sleep.^12^

The importance of sleep for healthy functioning and development in adolescence has been well researched. Inadequate sleep duration, later sleep timing, and sleepiness are significantly associated with a range of adverse health outcomes in adolescents such as poorer mental health, cognitive function, and school performance.^13^ Indeed, sleep has emerged as an important factor associated with mental health during adolescence, however the evidence base is largely cross‐sectional.^14^ In 2016, the first 24‐h movement guidelines were released in Canada which included recommendations for physical activity, sedentary behaviors, and sleep.^15^ For adolescents aged 14–17 years, it is recommended that 8–10 h of uninterrupted sleep per night should be attained.^15^ However, overall sleep health encompasses more than just total sleep duration, as it also encompasses sleep efficiency, sleep timing, subjective sleep quality, sleep latency, and alertness/sleepiness during waking hours.^16^

Physical activity is also known to influence components of sleep health including sleep duration, sleep latency, sleep efficiency and daytime sleepiness in youth.^17^ In a meta‐analysis examining the relationship between regular physical activity and sleep in adolescents and young adults (aged 14–24), a large, pooled effect was observed (ES = 0.89).^18^ However, given that the majority of included studies were cross‐sectional, causation cannot be determined and reverse causation is a possibility (i.e., sleep contributes to improvements in physical activity).^18^ Therefore, there is need for more experimental research examining the effects of physical activity interventions on sleep‐related variables, particularly in adolescents where research is lacking.^19^ Further, changes in sleep may also mediate changes in mental health as a result of physical activity, however more research is needed to support this hypothesis. To the best of our knowledge, no previous experimental studies have examined the mediating effects of sleep on older adolescents' mental health in the context of a physical activity intervention. The authors of a recent Lancet publication on adolescent health recommended that mediation studies should be conducted.^6^ Indeed, mediation analyses of randomized trials can provide new evidence about the mechanisms by which interventions may influence health outcomes.

Therefore, our study aims to address a gap in the literature by examining the potential mediating effects of dimensions of sleep health (sleep duration, sleep latency, awakenings, and daytime sleepiness), on older adolescents' mental health (perceived stress and internalizing disorders), in the context of a physical activity intervention. We will also examine the effects of a school‐based high‐intensity physical activity intervention on mental health outcomes in older adolescents, in comparison to a wait‐list control. We hypothesized that improvements in sleep‐related variables would mediate the effects of the physical activity intervention on mental health, and older adolescents in the intervention group would improve their mental health relative to the control group.

## 2 MATERIALS AND METHODS

### 2.1 Study design

Data for the current study were gathered from a subset of participants from the Burn 2 Learn (B2L) cluster randomized controlled trial (RCT).^20^ Detailed descriptions of the study protocol^21^ and findings^20^, ^22^, ^23^ have been reported previously. The trial was prospectively registered with the Australian and New Zealand Clinical Trials Registry (ACTRN12618000293268), and ethics approval was obtained from the Institutional Review Board of the University of Newcastle (H‐2016‐0424) and NSW Department of Education (SERAP: 2017116) human research ethics committees. The present study followed the AGReMA (A Guideline for Reporting studies of Mediation Analyses) reporting guidelines.

### 2.2 Sub‐study schools

In the larger trial, a 2‐arm parallel group RCT with an intervention group and wait‐list control group was conducted in 20 secondary schools in the Hunter, Central Coast, and Sydney regions in New South Wales (NSW), Australia. The RCT was conducted in two cohorts, with cohort 1 (10 schools; 5 × intervention, and 5 × control) commencing in 2018 and finishing in 2019, and cohort 2 (10 schools; 5 × intervention and 5 × control) commencing in 2019 and finishing in 2020. As sleep data were not collected in Cohort 1, the current study only includes participants from Cohort 2. Assessments for the current study were conducted at baseline (February–April, 2019), and immediately following the intervention period [approximately 6‐months from baseline (August–September, 2019)].

### 2.3 Participants

**FIGURE 1:**
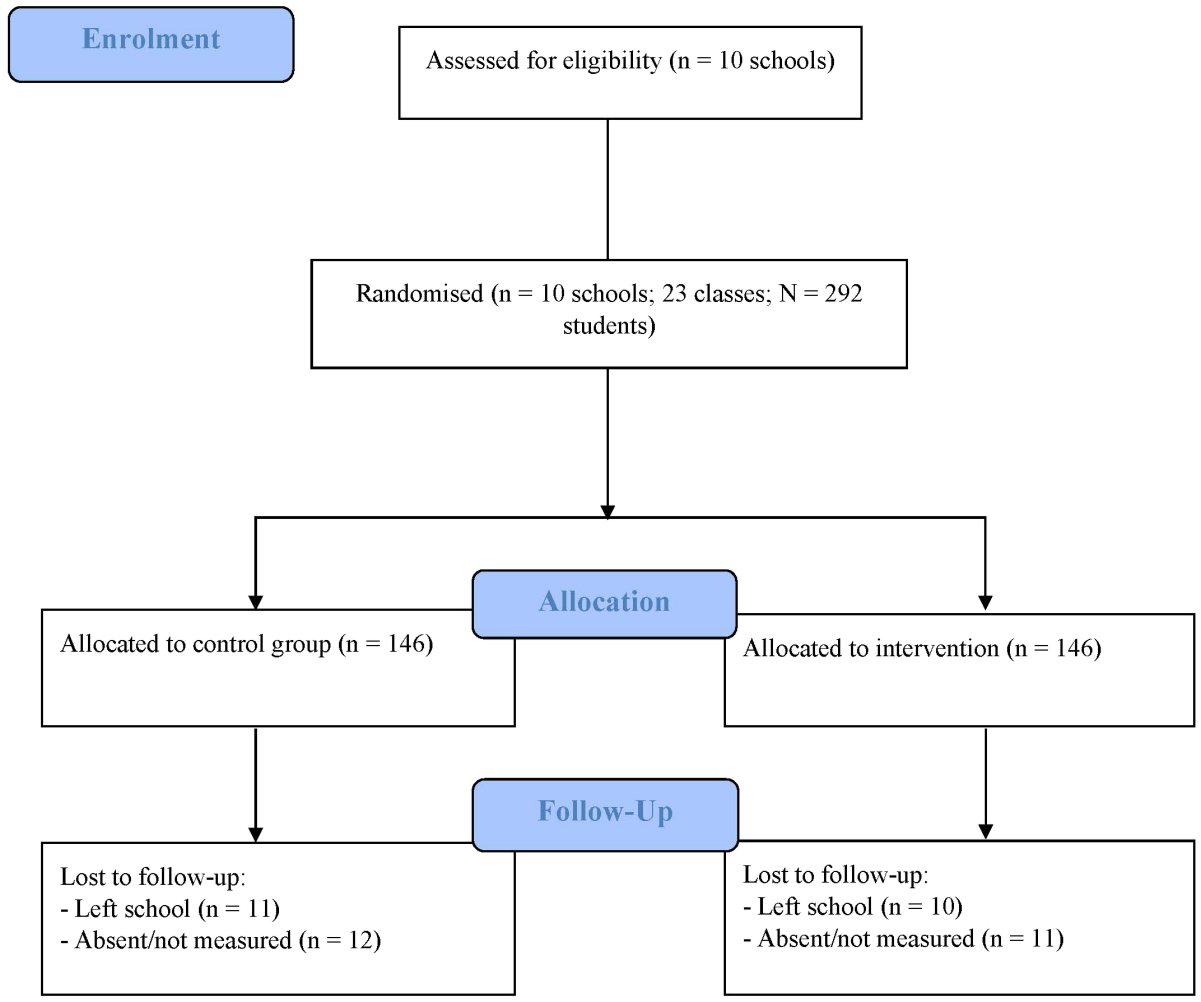
CONSORT flow diagram.

To be eligible for participation, study schools had to be government schools located within 150‐min drive from the University of Newcastle (e.g., Hunter‐Central Coast, Sydney, Northern Sydney, and Western Sydney regions). We recruited two Grade 11 teachers from each school who were willing to facilitate intervention delivery (there was no restriction placed on the teachers' subject discipline). Eligible participants were older adolescents in Grade 11 (15–17 years at baseline) taught by one of the participating teachers. A total of 292 older adolescents participated in the current study (Figure 1). School principals, teachers, parents, and students all provided written informed consent prior to enrolment.

### 2.4 Randomization

Following the completion of baseline data collection, schools were pair‐matched and then randomized by an independent researcher using a computer‐based random number generator. Schools were matched based on the following characteristics: geographic location, school area‐level socioeconomic status, and where possible, the teaching discipline of the participating class (e.g., Mathematics, English). One school from each pair was allocated to the B2L intervention and the other to the control condition (continued usual practice).

### 2.5 Sample size

The original power calculation was conducted to determine the sample size needed to detect an effect in the primary outcome of the larger cluster RCT (i.e., cardiorespiratory fitness). Baseline post‐test correlation (*r* = 0.90) and SD (29 laps) values were obtained from our pilot data,^24^ and intraclass correlation coefficient (ICC) values of 0.20 and 0.03 were used to account for clustering at the class‐levels and school‐levels, respectively.^25^

### 2.6 Intervention

A full description of the B2L intervention can be found in our published protocol paper.^21^ Briefly, B2L was a school‐based physical activity intervention designed for older adolescents. Teachers were provided with training, resources, and support to deliver 2 weekly high‐intensity physical activity breaks (hereafter referred to as B2L sessions) during academic lessons for 16 weeks. A 2‐week school holiday period occurred following week 10 of intervention delivery where B2L sessions were not delivered (sessions resumed once students returned from the school holiday break). B2L sessions involved a combination of aerobic (e.g., shuttle runs, jumping jacks, boxing) and body weight resistance (e.g., push‐ups, squat jumps, lunges) exercises, delivered in the form of high‐intensity interval training (HIIT). The typical length of B2L sessions was 10 min (including a brief warm‐up and cool‐down). Students were encouraged to reach a target intensity of 85% of their age‐predicted maximum heart rate, with exercise intensity monitored using heart rate monitoring technology (Wahoo TICKR), which paired with a purpose‐built B2L app to display students' heart rate.

### 2.7 Study measures

All assessments were conducted at the study schools by trained members of the research team. Demographic information [including socioeconomic status (SES)] and self‐report measures were obtained via an online survey administered using electronic tablets. SES was determined by population tertile using socioeconomic indexes for areas of relative socioeconomic disadvantage based on residential postcode. Anthropometric assessments were conducted privately (i.e., out of the view of other students) by same‐sex research staff, where possible.

#### 2.7.1 Intervention measures—Mental health constructs

*Perceived stress*: Participants completed the 10‐item perceived stress scale.^26^ For each item, participants responded using a 5‐point scale (i.e., “*never*” = 0, “*almost never*” = 1, “*sometimes*” = 2, “*fairly often*” = 3, “*very often*” = 4), in relation to the last month. The sum of all items was used to provide a measure of perceived stress (Chronbach's α = 0.83). A higher score indicates a greater degree of subjective stress experienced by participants.

*Internalizing problems*: The internalizing problems subscale consisted of 10 items (Chronbach's α = 0.70), from the Strengths and Difficulties Questionnaire.^27^ Items relating to ‘emotional’ and ‘peer problems’ (both include five items) were combined to provide a measure of internalizing problems. For each item, participants responded using a 3‐point scale (i.e., “not true” = 0, “somewhat true” = 1, “certainly true” = 2). Higher scores indicate more internalizing problems. Mental health classifications were as follows: for scores between 1 and 8, participants were classified as ‘close to average or slightly raised’, while participants with scores of 9–20 were classified as ‘high to very high’.^27^

#### 2.7.2 Hypothesized mediators—Sleep parameters

Several domains of sleep health were self‐reported by participants. For each domain, participants were required to reflect on the previous 2 weeks.

*Sleep duration*: Participants reported the time they usually went to bed and woke up on school days (weekdays) and weekends using a questionnaire.^28^ The difference between bed and wake times were calculated separately for weekdays and weekends and reported as total sleep duration in minutes.

*Sleep latency*: Participants responded to the following statement to provide a measure of sleep latency: “In the last two weeks, after you go to bed, how long does it usually take you to fall asleep?” Responses were scored on a 5‐point scale (i.e., “0–15 mins” = 1, “16–30 mins” = 2, “31–45 mins” = 3, “46–60 mins” = 4, “≥61 mins” = 5).^29^ A higher score indicates poorer sleep health.

*Awakenings*: Sleep awakenings refer to the number of episodes, per night, in which an individual is awake for greater than 5 min.^29^ Participants responded to the following statement to provide an indication of the number of sleep awakenings usually occurring per night: “In the last two weeks, how many times a night do you usually wake up at night for longer than five minutes?” Responses were scored on a 5‐point scale (i.e., “never” = 1, “once” = 2, “2 times” = 3, “3 times” = 4, “more than 3 times” = 5).^29^ A higher score indicates poorer sleep health.

*Daytime sleepiness*: Participants responded to the following statement to provide an indication of daytime sleepiness, which was adapted from the Children's Report of Sleep Patterns – Sleepiness Scale: “People sometimes feel sleepy during the daytime. During your daytime activities, how much of a problem do you have with sleepiness (feeling sleepy, struggling to stay awake)?”.^30^ Responses were scored on a 5‐point scale (i.e., “not a problem at all” = 1, “a little problem” = 2, “more than a little problem” = 3, “a big problem” = 4, “a very big problem” = 5). A higher score indicates higher daytime sleepiness.

### 2.8 Data analysis

**FIGURE 2:**
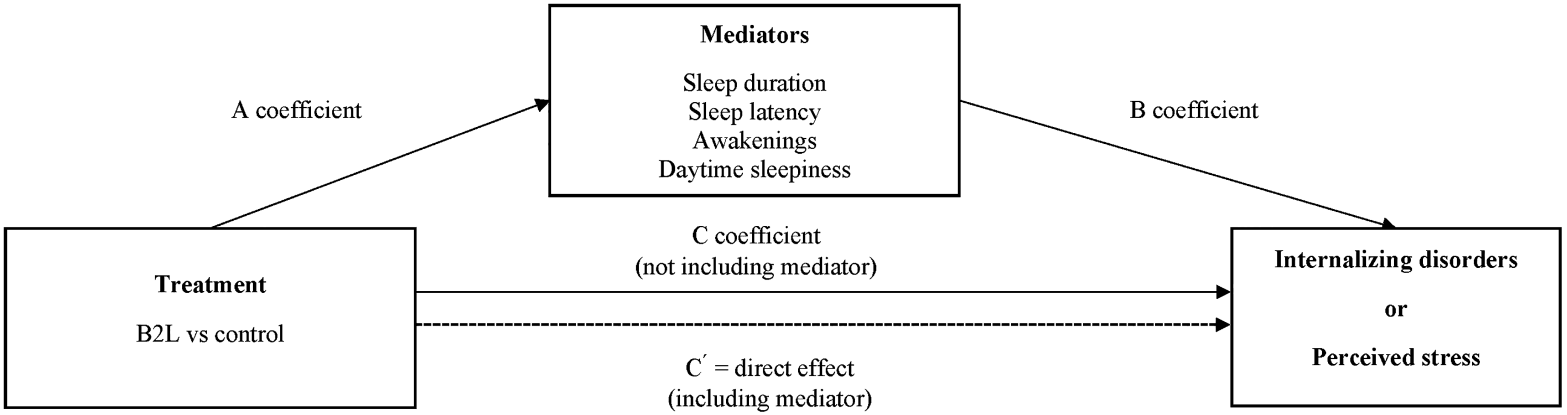
Mediation pathway for single mediator models; internalizing disorders and perceived stress.

Analyses were performed using the Mplus (Version 8.8; Múthen & Múthen). Means and standard deviations were calculated for all normally distributed variables. Bivariate correlations were used to examine correlations between variables (alpha levels set at *p* < 0.05). The robust maximum likelihood estimation procedure was used to account for missing data and the non‐independence of students nested within schools by adjusting the standard errors using a sandwich estimator. Linear regression models were used to test the mediation effects. The models were tested in the following steps with all models adjusted for baseline values of outcomes and the following covariates: sex, age, SES, and weight status at baseline. First, the total effect of the treatment (i.e., intervention versus control) on mental health outcomes at 6 months (perceived stress and internalizing problems) was examined separately (C pathway in Figure 2). In the second step, separate single‐mediator models were estimated to explore evidence for mediation effects. A multiple mediator analysis could not be conducted due to inadequate power. These models generated standardized regression coefficients (*β*) for: (i) the effect of the intervention on the mediators (i.e., weekday/weekend sleep duration, sleep latency, awakenings, and daytime sleepiness) (A pathways), (ii) the association between the mediators and mental health outcomes at 6 months (B pathways), and (iii) the direct effect of the intervention on perceived stress and internalizing problems independent of the mediated effect (C ´ pathway). The models also calculated the significance of the product‐of‐coefficients (A × B), which was used to determine the presence of an indirect effect. The indirect effect was considered statistically significant if the confidence intervals for the product‐of‐coefficients did not cross zero.

## 3 RESULTS

Demographic information for participants is presented in Table 1. In summary, most participants were born in Australia (80.8%), spoke English at home (88%), and parents were born in Australian (54.8%). In the current study, 28% of the participants were classified as overweight or obese, and 20% of participants reported ‘high to very high’ internalizing problems at baseline, which was similar to participants in cohort 1 (28% were classified as overweight or obese, and 18% reported ‘high to very high’ internalizing problems). Results of mediator models are displayed in Tables 2 and 3.

**TABLE 1:** Characteristics of study sample.

Characteristics	Total (*n* = 292)	B2L (*n* = 146)	Control (*n* = 146)
Age, mean (SD), years	16.0 (0.5)	16.0 (0.5)	16.0 (0.5)
Female participants, *n* (%)	143 (49.0)	82 (56.2)	61 (41.8)
Born in Australia, *n* (%)^a^	235 (80.8)	126 (86.3)	109 (75.2)
English spoken at home, *n* (%)	257 (88.0)	124 (84.9)	133 (91.1)
*Cultural background, n* (*%*)			
Australian	160 (54.8)	78 (53.4)	82 (56.2)
European	36 (12.3)	21 (14.4)	15 (10.3)
African	5 (1.7)	3 (2.1)	2 (1.4)
Asian	31 (10.6)	20 (13.7)	11 (7.5)
Middle Eastern	6 (2.1)	4 (2.7)	2 (1.4)
Other	54 (18.5)	20 (13.7)	34 (23.3)
Indigenous decent, *n* (%)	24 (8.2)	13 (8.9)	11 (7.5)
*Socioeconomic status, n* (*%*)^b^			
Low	52 (17.8)	28 (19.2)	24 (16.4)
Medium	96 (32.9)	61 (41.8)	35 (24.0)
High	144 (49.3)	57 (39.0)	87 (59.6)
*Mental health status, n* (*%*)^c^			
Close to average and slightly raised	231 (79.4)	119 (82.1)	112 (76.7)
High to very high	60 (20.6)	26 (17.9)	34 (23.3)
*Weight status, n* (*%*)^d^			
Underweight	15 (5.1)	5 (3.5)	10 (6.9)
Healthy weight	189 (64.7)	100 (69.9)	89 (61.8)
Overweight	55 (18.8)	24 (16.8)	31 (21.5)
Obese	28 (9.6)	14 (9.8)	14 (9.7)
*Sleep duration, mean* (*SD*)*, mins*			
Weekday	476.9 (71.9)	477.4 (70.2)	476.4 (73.7)
Weekend	524.2 (96.0)	528.9 (89.4)	519.4 (102.2)
*Sleep latency, n* (*%*)			
<15 mins	135 (46.6)	65 (45.1)	70 (47.9)
16–30 mins	82 (28.1)	46 (31.9)	36 (24.7)
31–45 mins	32 (11.0)	15 (10.4)	17 (11.6)
46–60 mins	24 (8.3)	11 (7.6)	13 (8.9)
61+ mins	17 (5.9)	7 (4.9)	10 (6.8)
*Awakenings, n* (*%*)			
Never	116 (41.6)	59 (41.5)	57 (41.6)
Once	84 (30.1)	44 (31.0)	40 (29.2)
2 times	46 (16.5)	23 (16.2)	23 (16.8)
3 times	11 (3.9)	6 (4.2)	5 (3.6)
More than three times	22 (7.9)	10 (7.0)	12 (8.8)
*Daytime sleepiness, n* (*%*)			
No problem	53 (18.3)	25 (17.4)	28 (19.2)
A little problem	152 (52.4)	75 (52.1)	77 (52.7)
More than a little problem	50 (17.2)	27 (18.8)	23 (15.8)
A big problem	23 (7.9)	12 (8.3)	11 (7.5)
A very big problem	12 (4.1)	5 (3.5)	7 (4.8)

^a^ One participant did not provide data for country of birth.

^b^ Socioeconomic status determined by population tertile using Socio‐Economic Indexes for Areas of relative socioeconomic disadvantage based on residential postcode.

^c^ One participant did not provide data for mental health status.

^d^ Five participants were not measured for weight status.

**TABLE 2:** Single‐mediator models explaining perceived stress.

	Intervention on mediator			Mediator on outcome			Intervention on outcome			Mediated effect		
Variables	A (β)	95% CI	*p*	B (β)	95% CI	*p*	C (β)	95% CI	*p*	AB (SE)	95% CI	*p*
Sleep duration (WD)	−4.50 (−0.06)	−19.62, 10.62	0.560	−0.09 (−0.11)	−0.18, −0.00	0.046	−1.02 (−0.16)	−2.07, 0.03	0.056	0.04 (0.07)	−0.10, 0.18	0.550
Sleep duration (WE)	9.89 (0.11)	−9.56, 29.34	0.319	−0.06 (−0.09)	−0.15, 0.02	0.150	−0.86 (−0.14)	−1.85, 0.13	0.089	−0.06 (0.09)	−0.23, 0.11	0.471
Sleep latency	−0.02 (−0.02)	−0.24, 0.20	0.867	0.51 (0.09)	0.01, 1.02	0.046	−0.95 (−0.15)	−1.98, 0.09	0.073	−0.01 (0.06)	−0.12, 0.11	0.871
Awakenings	−0.06 (−0.05)	−0.29, 0.18	0.644	0.51 (0.09)	0.03, 0.98	0.037	−1.08 (−0.18)	−2.12, −0.03	0.043	−0.03 (0.06)	−0.15, 0.10	0.661
Daytime sleepiness	−0.17 (−0.19)	−0.36, 0.02	0.075	1.13 (0.17)	0.46, 1.79	0.001	−0.85 (−0.14)	0.56, 0.72	0.125	−0.19 (0.13)	−0.44, 0.06	0.131

*Note*: A, unstandardized regression coefficient for treatment condition predicting mediators at 6 months; AB, product‐of‐coefficients estimate; B, unstandardized regression coefficient for mediators at 6 months predicting perceived stress at 6 months; Ć, unstandardized regression coefficient for treatment condition predicting perceived stress at 6 months with adjustment for mediators, otherwise known as the direct effect; SE, standard error; β, standardized regression coefficients.

**TABLE 3:** Single‐mediator models explaining internalizing disorders.

	Intervention on mediator			Mediator on outcome			Intervention on outcome			Mediated effect		
Variables	A (β)	95% CI	*p*	B (β)	95% CI	*p*	C ´ (β)	95% CI	*p*	AB (SE)	95% CI	*p*
Sleep duration (WD)	−4.37 (−0.06)	−19.55, 10.81	0.573	−0.04 (−0.09)	−0.07, −0.01	0.019	0.27 (0.09)	−0.36, 0.90	0.401	0.02 (0.31)	−0.05, 0.08	0.357
Sleep duration (WE)	9.96 (0.11)	−9.50, 29.43	0.316	−0.01 (−0.04)	−0.04, 0.02	0.406	0.37 (0.12)	−0.25, 0.99	0.236	−0.01 (0.02)	−0.06, 0.03	0.569
Sleep latency	−0.02 (−0.02)	−0.24, 0.20	0.867	0.25 (0.09)	−0.11, 0.61	0.170	0.30 (0.09)	−0.33, 0.93	0.352	−0.01 (0.03)	−0.06, 0.05	0.875
Awakenings	−0.05 (−0.05)	−0.29, 0.19	0.670	−0.10 (−0.04)	−0.37, 0.17	0.464	0.24 (0.08)	−0.39, 0.88	0.455	0.01 (0.01)	−0.02, 0.03	0.656
Daytime sleepiness	−0.17 (−0.19)	−0.36, 0.02	0.075	0.67 (0.19)	0.38, 0.95	<0.001	−0.33 (0.11)	−0.36, 0.02	0.300	−0.11 (0.07)	−0.24, 0.02	0.083

*Note*: A, unstandardized regression coefficient for treatment condition predicting mediators at 6 months; AB, product‐of‐coefficients estimate; B, unstandardized regression coefficient for mediators at 6 months predicting internalizing disorders at 6 months; Ć, unstandardized regression coefficient for treatment condition predicting internalizing disorders at 6 months with adjustment for mediators, otherwise known as the direct effect; SE, standard error; β, standardized regression coefficients.

### 3.1 Intervention effect on mental health

We observed a small significant reduction in perceived stress in favor of the intervention group (*β* = −0.11, SE = 0.034, *p* = 0.002). No significant effects were observed for internalizing problems (*β* = 0.02, SE = 0.051, *p* = 0.760).

### 3.2 Intervention effect on potential mediators

There were no significant intervention effects on any sleep‐related variables, after adjusting for covariates. However, there was a marginally significant intervention effect on daytime sleepiness in the hypothesized direction (*β* = −0.19, SE = 0.052, *p* = 0.075).

### 3.3 Mediator effects on mental health outcomes

After adjusting for covariates, weekday sleep duration was negatively associated with perceived stress (*β* = −0.11, SE = 0.058, *p* = 0.046) and internalizing problems (*β* = −0.09, SE = 0.038, *p* = 0.019). Daytime sleepiness was positively associated with perceived stress (*β* = 0.17, SE = 0.049, *p* = 0.001) and internalizing problems (*β* = 0.019, SE = 0.042, *p* < 0.001). Sleep latency (*β* = 0.09, SE = 0.044, *p* = 0.046) and awakenings (*β* = 0.09, SE = 0.04, *p* = 0.037), were both positively associated with perceived stress.

### 3.4 Significance of the indirect effects

None of the sleep‐related variables satisfied the criteria for mediation.

## 4 DISCUSSION

The primary aim of our study was to explore the mediating effect of sleep‐related variables on older adolescents' mental health, in the B2L cluster RCT. To our knowledge, this is the first experimental study to examine the mediating effect of sleep‐related variables on mental health outcomes in older adolescents in the context of a physical activity intervention. No overall mediation effects were found, despite mediators being associated with mental health outcomes. Our analyses showed that the B2L intervention had a significant effect on adolescents' perceived stress, however, there were no significant effects on internalizing problems. The B2L intervention did not significantly improve any sleep‐related mediators, although the effect on daytime sleepiness approached significance.

Contrary to our hypothesis, we did not observe an indirect effect of a physical activity intervention on mental health via sleep (i.e., behavioral pathway). Therefore, the intervention effect we observed for perceived stress was likely caused by some other mechanism. One possible explanation is that the B2L intervention reduced participants' perceived stress via a neurobiological pathway.^12^, ^31^ According to the cross‐stressor adaptation (CSA) hypothesis,^32^ participation in physical activity of sufficient intensity may moderate the functioning of underlying stress physiology, resulting in a stress buffering effect. Specifically, physical activity may lead to a less pronounced physiological stress response [e.g., reduced cardiovascular response (e.g., heart rate and blood pressure) and blunted cortisol secretion], by influencing the activation of the hypothalamic–pituitary‐adrenocortical axis and sympathoadrenal medullary system.^32^ While this was unable to be measured in the current study, we previously examined hair cortisol concentration as a biomarker of chronic exposure to stress among participants in the first cohort of our larger RCT (cortisol samples were not collected in the second cohort).^20^ Consistent with the CSA hypothesis, we observed a significant effect for hair cortisol concentration in favor of the intervention group. While it is plausible that the effect of physical activity on mental health may have been indirectly affected via this neurobiological pathway, it is also plausible to suggest other neurobiological mechanisms (e.g., myokines) or psychosocial (e.g., needs satisfaction) may have contributed to this effect. As such, future studies with rigorous research designs are needed to confirm these findings.

Given the increase in mental health issues (i.e., stress, anxiety, depression) experienced by adolescents in recent years,^2^ it is promising to observe that participating in brief physical activity breaks during the academic lessons led to a reduction in adolescents' perceived stress. Previous research examining the effects of school‐based physical activity interventions on measures of stress in adolescent populations has been mixed.^33^ In the scoping review conducted by Pascoe and colleagues,^33^ yoga interventions did not significantly decrease stress in comparison to regular physical education lessons, however, an aerobic physical activity intervention (of vigorous intensity) significantly decreased adolescents' stress in comparison to a moderate‐intensity, flexibility, and control group. While it is difficult to compare intervention effects due to differences in control conditions, physical activity intensity may play a key role in providing mental health benefits.^12^ For example, evidence has demonstrated that vigorous intensity physical activity is particularly effective for reducing stress and ill‐being symptoms.^34^, ^35^

Contrary to the effect on stress, the B2L intervention did not significantly reduce symptoms of internalizing problems among adolescents. While physical activity is known to benefit mental health, it is expected that effects are more pronounced among populations deemed to be at risk of poor mental health. For example, in a review conducted by Marker and colleagues,^36^ the overall effect on well‐being was only marginally significant when one study examining the effects of a Pilates intervention in children with idiopathic arthritis was removed from the analysis. This is consistent with findings from a recent meta‐analysis, which found that effects for reducing internalizing symptoms (i.e., anxiety), was larger in clinical compared to non‐clinical populations.^37^ In the current study, only one in five participants reported experiencing high levels of internalizing problems, which may partly explain why an effect was not observed among the full study sample (i.e., potential floor effect). Taken together, these findings highlight that physical activity interventions are likely to be more effective for participants at risk of poor mental health.

The B2L intervention did not have a significant effect on any of the sleep‐related variables. Prior research demonstrates that acute and chronic physical activity interventions can improve sleep outcomes in adult populations,^38^ but adolescent studies are lacking. Indeed, a recent review identified only two experimental studies examining the effects of physical activity interventions among adolescents, with both studies reporting improvements in sleep outcomes following the intervention period.^19^ Notably, the included studies incorporated similar physical activity interventions (i.e., moderate‐vigorous intensity aerobic activity, delivered 5 days per week), which is in contrast to our intervention (i.e., vigorous‐intensity activity, delivered 2 days per week) and may partly explain the different study findings. At present, there is insufficient evidence among adolescents to determine the optimal dose of physical activity needed to improve sleep outcomes, however, there is some support for high frequency, moderate‐intensity physical activity.^19^ To gain a greater understanding of how physical activity can benefit adolescents' sleep, more high‐quality RCTs that examine varying quantitative aspects of physical activity (e.g., frequency, intensity, time, and type) are needed.^19^

The effect on daytime sleepiness was also not significant in our study. In our previous sub‐study we found that a single session of B2L led to significant improvements in students' subjective vitality (i.e., increased energy, alertness, and aliveness).^22^ It is plausible that subjective vitality and daytime sleepiness are overlapping constructs, whereby a change in one construct is likely to influence the other (i.e., higher levels of vitality would be expected with lower levels of sleepiness and vice versa). One possible explanation for the lack of effect observed in the current study is that regular HIIT sessions might influence daytime sleepiness by providing adolescents with higher quality sleep, but it is equally (and perhaps more) plausible that an acute HIIT session reduces feelings of sleepiness through the activating effects of exercise (as implied by the findings of our acute study). In this context, HIIT may be acting as a short‐term stimulant, rather than a prophylactic for poor quality sleep. Indeed, the null effect on awakenings, another marker of sleep quality, supports this interpretation. The associations between sleep, physical activity and mental health are complex, bidirectional, and interacting, and so explanations for associations between them are difficult even with data from experimental studies. That there was an effect on adolescents' mental health that did not operate via effects on sleep raises further interesting questions as to what mechanism was operating to achieve this effect.

Although B2L did not improve measures of sleep, we observed several statistically significant associations with sleep variables and mental health in the hypothesized directions. These findings are consistent with observational evidence demonstrating the importance of sleep for mental health in children and adolescents.^14^ Despite a lack of an intervention effect on sleep variables, it is still of interest to observe these associations between sleep and mental health outcomes, as it highlights a potential pathway by which improvements in mental health may be achieved. As such, interventions that can improve measures of sleep among adolescents, may be an effective strategy for improving mental health among adolescents, however, further experimental evidence is needed. While physical activity and good sleep hygiene have been shown to promote better sleep, other factors such as technology use, caffeine intake, and early school start times have been shown to inhibit sleep quality.^39^ For example, research in adolescents has shown that sleep problems (e.g., problems falling asleep/staying asleep and short sleep duration) mediated the association between technology use and depressive symptoms.^40^ As such, interventions that target multiple behaviors are likely to be more effective in improving measures of sleep than physical activity alone.

### 4.1 Strengths and limitations

The strengths of our study include the robust cluster RCT design, unique study population, adherence to the AGReMA reporting guidelines for mediation analysis, and examination of novel mediators. There are some limitations that should also be acknowledged. First, the study was underpowered to detect small changes in mental health and sleep‐related measures. As mentioned previously, our study was powered to detect change in the primary outcome of the larger cluster RCT (i.e., cardiorespiratory fitness) and we did not measure sleep variables in the first study cohort. Second, all measures for mental health outcomes and hypothesized mediators were self‐reported, therefore the influence of social desirability bias cannot be discounted. Further, sleep measures were single item questionnaire and may not have been sensitive enough to detect change. Objective monitoring of sleep, for example using polysomnography or actigraphy, may have produced more accurate estimates for some sleep parameters. It is important to note that sleep duration was analyzed as a continuous variable. This could be problematic because sleep duration above the target range (e.g., sleep in excess of 10 h) is not a good indicator of sleep quality. Of note, fewer than 2% of participants reported more than 10 h sleep on weekdays; however, a greater proportion of participants (approximately 10%) reported more than 10 h sleep on weekends. Finally, there were a small number of students who left school prior to the completion of the study (7.2%). As such, not all students provided follow‐up data. However, the percentage of students who left school was similar between intervention and control groups (6.8% and 7.5%), respectively.

## 5 PERSPECTIVE

This is the first study to examine the potential mediating effects of sleep‐related variables on older adolescents' mental health in the context of a school‐based physical activity intervention. Our study addresses a notable limitation of the current evidence base by exploring mechanisms underlying changes in mental health.^6^, ^12^ Our findings suggest that changes in sleep parameters did not mediate the intervention effects on older adolescents' mental health. Our study showed that participating in short duration HIIT twice per week, had a significant effect on perceived stress but not on internalizing problems. Given the lack of an indirect effect, this suggests that the intervention effect on stress was likely driven by other mechanisms. Future research should continue to explore the potential neurobiological, psychosocial, and behavioral mechanisms by which physical activity might influence mental health for the purposes of optimizing intervention effects.

## AUTHOR CONTRIBUTIONS

AAL: Data curation, methodology, resources, investigation, writing—original draft, writing—review and editing. TMOD: Data curation, formal analyses, investigation, writing review and editing. NE: Funding acquisition, methodology, resources, investigation, writing ‐ review and editing; MJD: methodology, investigation, writing—review and editing. JJS: Funding acquisition, methodology, resources, writing—review and editing. PJM: Funding acquisition, methodology, writing—review and editing. DRL: Conceptualization, funding acquisition, investigation, methodology, resources, supervision, writing—review and editing.

## FUNDING INFORMATION

The study was funded by the National Health and Medical Research Council (APP1120518) and the New South Wales Department of Education School Sport Unit. DRL is supported by a National Health and Medical Research Council Research Fellowship (APP1154507). MJD is supported by a Career Development Fellowship (APP1141606) from the National Health and Medical Research Council.

## CONFLICT OF INTEREST STATEMENT

The authors have no conflict of interest to declare.

## Data Availability

Data are available upon reasonable request. Requests for access to data from the study should be addressed to the corresponding author at angus.leahy@newcastle.edu.au. The study protocol has been published. All proposals requesting data access will need to specify how it is planned to use the data, and all proposals will need approval of the trial co‐investigator team before data release.
